# Sex and age effects on gray matter volume trajectories in young children with prenatal alcohol exposure

**DOI:** 10.3389/fnhum.2024.1379959

**Published:** 2024-04-10

**Authors:** Madison Long, Preeti Kar, Nils D. Forkert, Bennett A. Landman, W. Ben Gibbard, Christina Tortorelli, Carly A. McMorris, Yuankai Huo, Catherine A. Lebel

**Affiliations:** ^1^Department of Radiology, University of Calgary, Calgary, AB, Canada; ^2^Owerko Centre, Alberta Children Hospital Research Institute, University of Calgary, Calgary, AB, Canada; ^3^Hotchkiss Brain Institute, University of Calgary, Calgary, AB, Canada; ^4^Department of Electrical and Computer Engineering, Vanderbilt University, Nashville, TN, United States; ^5^Department of Computer Science, Vanderbilt University, Nashville, TN, United States; ^6^Department of Paediatrics, University of Calgary, Calgary, AB, Canada; ^7^Department of Child Studies and Social Work, Mount Royal University, Calgary, AB, Canada; ^8^Werklund School of Education, University of Calgary, Calgary, AB, Canada; ^9^Mathison Centre for Mental Health Research and Education, Calgary, AB, Canada

**Keywords:** prenatal alcohol exposure, structural MRI, gray matter volume, development, trajectories, teratogen, early childhood

## Abstract

Prenatal alcohol exposure (PAE) occurs in ~11% of North American pregnancies and is the most common known cause of neurodevelopmental disabilities such as fetal alcohol spectrum disorder (FASD; ~2–5% prevalence). PAE has been consistently associated with smaller gray matter volumes in children, adolescents, and adults. A small number of longitudinal studies show altered gray matter development trajectories in late childhood/early adolescence, but patterns in early childhood and potential sex differences have not been characterized in young children. Using longitudinal T1-weighted MRI, the present study characterized gray matter volume development in young children with PAE (*N* = 42, 84 scans, ages 3–8 years) compared to unexposed children (*N* = 127, 450 scans, ages 2–8.5 years). Overall, we observed altered global and regional gray matter development trajectories in the PAE group, wherein they had attenuated age-related increases and more volume decreases relative to unexposed children. Moreover, we found more pronounced sex differences in children with PAE; females with PAE having the smallest gray matter volumes and the least age-related changes of all groups. This pattern of altered development may indicate reduced brain plasticity and/or accelerated maturation and may underlie the cognitive/behavioral difficulties often experienced by children with PAE. In conjunction with previous research on older children, adolescents, and adults with PAE, our results suggest that gray matter volume differences associated with PAE vary by age and may become more apparent in older children.

## 1 Introduction

Brain development begins *in-utero* and is affected by the gestational environment. Prenatal alcohol exposure (PAE) is known to disrupt most fetal developmental processes (Randall and Taylor, 1979; Abel and Sokol, 1986; Jacobson et al., 1993; Goodlett et al., 2005; Sambo and Goldman, 2023) and can result in a wide variety of birth defects (Dyląg et al., 2023). Importantly, PAE affects fetal brain development on both molecular and cellular levels (Greene and Copp, 2014; Wilhelm and Guizzetti, 2016; Pinson et al., 2022; Adams et al., 2023; Boschen et al., 2023), with potential effects on brain structure detectable via magnetic resonance imaging (MRI) from birth through adulthood (Lebel et al., 2011; Donald et al., 2015, 2016). PAE has an estimated prevalence of 11% in North America (Popova et al., 2017). In some cases, PAE causes neurodevelopmental delays such as fetal alcohol spectrum disorder (FASD, ~2–5% prevalence; May et al., 2018), which makes it the most common known cause of neurodevelopmental delays in the United States and Canada. Children with PAE commonly experience neurodevelopmental difficulties across various domains, including executive functions, gross and fine motor skills, language, and visual perception (Mattson et al., 2011, 2019). Because early childhood is a period of rapid brain structural and neurodevelopmental change (Bethlehem et al., 2022), characterizing how brain development is altered in children with PAE can help to improve the understanding of the mechanisms underlying different neurodevelopmental and behavioral outcomes in children with PAE.

Previous MRI studies have found widespread structural brain differences in individuals with PAE as compared to unexposed individuals. This includes smaller total brain, white matter, and gray matter volume (see Lebel et al., 2011; Donald et al., 2015 for reviews). Smaller gray matter volumes have been reported for the caudate (Mattson et al., 1996; Archibald et al., 2001; Cortese et al., 2006; Astley et al., 2009; Nardelli et al., 2011), pallidum and putamen (Nardelli et al., 2011; Roussotte et al., 2011; Treit et al., 2013), and the basal ganglia more broadly (Mattson et al., 1996), as well as the hippocampus (Willoughby et al., 2008; Astley et al., 2009; Nardelli et al., 2011; Treit et al., 2013), thalamus (Mattson et al., 1992; Nardelli et al., 2011; Treit et al., 2013), and frontal, temporal, and parietal cortex (Astley et al., 2009; Chen et al., 2012; Rajaprakash et al., 2014).

Limited longitudinal research has indicated that trajectories of gray matter volume development differ between individuals with and without PAE. For example, Lebel et al. (2012) showed significant differences in gray matter volume trajectories in posterior brain regions for individuals with PAE aged 5–20 years wherein individuals with PAE showed more linear trajectories, with a smaller magnitude of change over time, than the unexposed group. Treit et al. (2013) found a similar pattern, with fewer age related increases in cortical and deep gray matter volumes for individuals with FASD aged 5–15 years than controls. However, group differences did not reach statistical significance (Treit et al., 2013). Hendrickson et al. (2018) observed differences in 2-year cortical volume trajectories, primarily in right-hemispheric regions, in children and adolescents with PAE (Hendrickson et al., 2018). In all three studies, individuals with PAE showed a smaller magnitude of change with age than unexposed children (Lebel et al., 2012; Treit et al., 2013; Hendrickson et al., 2018). Cross-sectional studies report more mixed findings, showing more curvilinear trajectories and greater volume decreases in the PAE group (Inkelis et al., 2020), or no difference between groups (Nardelli et al., 2011; Rajaprakash et al., 2014; Zhou et al., 2018). This highlights the importance of longitudinal designs to connect findings from samples of different age ranges, as well as to measure true within-subject development trajectories to estimate the magnitude and direction of group-level changes.

PAE has also been associated with sex differences in brain volume, neurogenesis, and hypothalamic–pituitary–adrenal (HPA) axis function (see reviews by Terasaki et al., 2016 and Weinberg et al., 2008). With respect to brain structure in children with PAE, sex appears to play an important role. For example, Nardelli et al. (2011) found fewer regional volume differences between males and females with PAE as compared to an unexposed group, where males had larger gray matter than females. Others have found that the magnitude of volume reductions in samples with PAE vary by sex. Treit et al. (2017) found males with PAE had 12–17% smaller volume in the thalamus, putamen, and caudate than unexposed males, whereas females with PAE had 7–12% smaller volumes than unexposed females (Treit et al., 2017). Similarly, Subramoney et al. (2022) found that males with PAE had significantly smaller putamen volume than unexposed males while females from both groups had similar putamen volume (Subramoney et al., 2022). There are no major sex differences in the types of FASD diagnosis (i.e., FASD with sentinel facial features versus FASD without sentinel facial features) or the physical characteristics of FASD, but the prevalence of various mental health and cognitive outcomes varies for males and females with PAE. While no sex differences were noted in the preschool years, male children, adolescents, and adults with PAE show higher rates of ADHD, conduct disorder, oppositional defiant disorder, and motor, memory, attention, executive function, and adaptive functioning impairment, while females show higher rates of anxiety and depressive/mood disorders (Flannigan et al., 2023). Thus, further examinations of sex differences in the brain in individuals with PAE may elucidate sex-specific neural characteristics underlying differential outcomes neurodevelopmental, behavioral, and mental health outcomes.

The present study aimed to characterize regional gray matter volume development using T1-weighted structural MRI in a longitudinal sample of young children with PAE aged 2.9–8.07 years and a similarly aged sample of unexposed children. We predicted overall smaller gray matter volumes in children with PAE across ages and smaller/fewer age-related changes in volume. Additionally, we predicted that the magnitude of volume reductions in the PAE group would vary by sex.

## 2 Methods

### 2.1 Participants

Children with PAE were recruited through caregiver support groups, early intervention services, and Alberta Children’s Services in Alberta, Canada. Exclusion criteria were birth before 34 weeks’ gestation, children for whom English was not a primary language, history of head trauma, a diagnosis of autism, cerebral palsy, epilepsy or any other medical or genetic disorder associated with a serious motor or cognitive disability, and contraindications to MRI (e.g., metal implants, dental braces). Children with neurodevelopmental disorders such as attention deficit hyperactivity disorder (ADHD), learning disabilities, language delays, and/or mental health diagnoses were included, as these diagnoses are frequently comorbid with PAE. Initial recruitment consisted of 57 children with confirmed PAE between ages 2 and 7 years. Children and their caregivers and were invited to return approximately every 6 months for a follow-up MRI scan. One child was excluded for an incidental finding on the MRI scan and 2 children did not feel comfortable receiving an MRI scan. Additionally, 12 of the collected T1-weighted MRI scans were excluded due to low quality, resulting in a data set of 42 subjects (50% female) with 84 scans (range 1–4 scans/subject) and a full age range of 2.9–8.07 years. In the final sample of subjects with PAE included in this study, 13 were scanned once, 17 were scanned twice, and 11 were scanned three times, and 1 subject was scanned four times. The average inter-scan interval was 0.7 years (range 0.5–1.6 years). The median household income of the PAE group was 75,000–99,999 CAD (59,700–79,600 USD). None of these participants were diagnosed with FASD at the time of recruitment. PAE was confirmed in all participants via the subject’s child welfare file, which contained information reported from birth families, social workers, police records, and medical files. and/or using a semi-structured interviews with current caregivers, caseworkers, and/or birth families. 31% of the exposed participants had confirmed PAE greater than or equal to the threshold in the Canadian Diagnostic Guidelines for FASD (Cook et al., 2016): ≥7 drinks in 1 week and/or two or more binge episodes (≥4 drinks at one time) during pregnancy; the remaining 69% had confirmed PAE of an unspecified amount. 93% of participants with PAE also had prenatal exposure to other substances. Specifically, 55% of subjects were also exposed prenatally to cocaine, 45% to cannabis, 36% to cigarettes, 19% to methamphetamine, 14% to opioids, 7% to benzodiazepines, and 5% had drug exposure of an unspecified type. Seventy subjects had one prenatal drug exposure in addition to alcohol, 10 subjects had two additional drug exposures, 8 subjects had three, 3 subjects had four, and 1 subject had 6 additional drug exposures. 3 subjects had no other reported prenatal drug exposure except for alcohol. Additionally, 74% (*n* = 40) had adverse postnatal experiences such as neglect, physical/sexual/emotional abuse, witnessing violence and/or substance use, and/or multiple caregiver transitions. The remaining 26% (*n* = 14) of participants with PAE had no known postnatal adverse exposures. None of the participants with PAE were residing with their biological parents; all were in adoptive, foster, or kinship care. The age of stable placement, after which there was an absence of reported postnatal adverse experiences (as defined above), ranged from 0 to 4.08 years.

The unexposed sample consisted of children in the Calgary Preschool MRI study (Reynolds et al., 2020), who were recruited from Calgary, Alberta and surrounding areas and from the ongoing Alberta Pregnancy Outcomes and Nutrition (APrON) study (Kaplan et al., 2014). Inclusion criteria were born >36 weeks’ gestation, spoken English as a primary language, no contraindications to MRI scans, and no history of developmental delays, presence of a neurodevelopmental disability or brain trauma. Unexposed participants had confirmed absence of PAE and prenatal exposure to other substances based on either prospective questionnaires and interviews completed with the mother during pregnancy (APrON subjects) or retrospective reports. Additionally, unexposed subjects had no reported postnatal adversities (i.e., abuse, neglect). A total of 450 high-quality scans from 127 unexposed children (50% female) were included. Children were recruited between ages 2 and 6 years and scanned at approximately 6-month intervals for a full age range of 1.9–8.4 years. The sample included two pairs of non-twin full-siblings. All unexposed participants were residing with their biological parent(s) at the time of their MRI scan. The median household income of the unexposed group was 100,000–124,999 CAD (79,600–99,600 USD). Demographic characteristics for both samples are listed in Table 1.

**Table 1:** Demographics for the unexposed group and group with PAE.

		Unexposed (%)	PAE (%)
Parent marital status	Single	1 (<1)	4 (10)
	Married/common law	120 (95)	22 (52)
	Divorced/separated	3 (2)	5 (12)
Maternal education	High School Diploma	1 (<1)	4 (10)
	Some postsecondary	5 (4)	7 (17)
	Trade/Technical Diploma	24 (19)	5 (12)
	Undergraduate degree	56 (44)	5 (12)
	Some post-graduate	1 (<1)	4 (10)
	Graduate degree	37 (29)	3 (7)
Family income	Less than 25,000	0	4 (10)
	25,000–49,999	3 (2)	5 (12)
	50,000–74,999	5 (4)	13 (31)
	75,000–99,999	25 (20)	7 (17)
	100,000–124,999	26 (21)	11 (26)
	125,000–149,999	5 (4)	0
	150,000–174,999	18 (14)	1 (2)
	175,000 and up	41 (33)	0
Other prenatal substance exposures	Cigarettes	0	15 (36)
	Cannabis	0	19 (45)
	Cocaine	0	23 (55)
	Opioids	0	6 (14)
	Benzodiazapines	0	3 (7)
	Methamphetamine	0	8 (19)
	Other drugs - unspecified	0	5 (12)

### 2.2 Magnetic resonance image acquisition

For all children, MRI scans were completed on a research-dedicated GE 3 T MR750w system with a 32-channel head coil at the Alberta Children’s Hospital. Families were given reading materials to prepare children at home and were offered one or more practice sessions in an MRI simulator (Thieba et al., 2018). To minimize head motion throughout the scan, foam padding was used, and children were able to watch a movie using headphones, a projector, and a screen. The MRI protocol included the acquisition of T1-weighted anatomical images (FSPGR BRAVO sequence parameters: 0.9 × 0.9 × 0.9 mm resolution, 210 axial slices, TR = 8.23 ms, TE = 3.76 ms, flip angle = 12 degrees, matrix size = 512×512, inversion time = 540 ms). During T1 acquisition, children were not sedated and were awake viewing a movie of their choice or sleeping naturally.

### 2.3 Image processing

Images were initially assessed for quality at the scanner at the time of acquisition, and sequences were repeated if necessary and if time permitted. Images were also examined for motion after acquisition; those with major motion artifacts were excluded. During processing, N4 bias corrected images (Tustison et al., 2010) were resampled to a voxel size of 1 mm in preparation for multi-atlas segmentation combined with cortical reconstruction using implicit surface evolution (MaCRUISE; Huo et al., 2016a,b, 2018). MaCRUISE integrates the processes of cortical reconstruction and multi-atlas segmentation to produce reliable and consistent cortical surface parcellations in anatomical agreement with brain segmentations (Huo et al., 2016a,b, 2018). In the MaCRUISE pipeline, skull and dura-stripped images are subject to both multi-atlas segmentation of 132 regions (Klein et al., 2010; Asman and Landman, 2012, 2013) and TOpology-preserving Anatomical Segmentation (TOADS) fuzzy membership segmentation (Bazin and Pham, 2008). MaCRUISE then fuses the rigid multi-atlas and TOADS segmentations, resulting in a full cerebrum segmentation comprised of a gray matter and white matter component. To achieve a cortical reconstruction consistent with the segmentations, MaCRUISE applies multi-atlas anatomically consistent gray matter enhancement (MaACE; Sethian, 1999; Han and Fischl, 2007) to the gray matter component while applying a topology correction to the white matter component (Han et al., 2001, 2002). These refined gray and white matter segmentations form the outer and inner surfaces of the reconstructed cortex, respectively. Lastly, to resolve any remaining disagreements between the multi-atlas segmentation and reconstructed surfaces, MaCRUISE refines boundaries in the MA segmentation using the inner and outer cortical surfaces (Huo et al., 2016b). We extracted the refined segmentations (in ml) for analyses of regional volume. After automatic segmentation, trained raters checked the segmentations for accuracy and assigned them a quality score of 1 (poor), 2 (unsatisfactory), 3 (satisfactory), and 4 (excellent). Segmentations with a quality score < 3 were manually edited and reintroduced to the MaCRUISE pipeline at the segmentation fusion step, in place of the original rigid multi-atlas segmentation. Edited segmentation outputs were reassessed for quality and included in the analysis if the resulting segmentation obtained a quality score of 3 or 4. At each of the quality assessment stages, raters were blinded to the group membership of individual subjects in order to reduce bias. In total, 87% of the unexposed group scans and 81% of scans from the PAE group were retained for analysis. Lastly, we performed a longitudinal registration of the data to better ensure the biological plausibility of volume change between timepoints and prevent additive effects of small segmentation errors on the longitudinal analysis. Therefore, the T1-weighted image from the last/oldest timepoint was registered to each previous timepoint employing a non-linear transformation using NiftyReg software (NiftyReg—CMIC, 2023). Next, the resulting non-linear transformation fields were applied to the segmentation image for the oldest timepoint, warping the final segmentation to the image space of each previous timepoint. Final volume measurements were computed in the original T1 image space for each timepoint. Spaghetti plots of the final volume values were visually inspected to ensure plausibility of inter-scan volume changes and identify outlying values. We identified one participant who consistently had very small volume values. Analyses were run both with and without this participant included and results were unchanged.

### 2.4 Statistical analysis

To characterize volume development trajectories, we compared a series of mixed effects models for each of the 116 gray matter regions, as well as total ICV and total gray matter volume and selected the one with the best fit as determined by the Akaike Information Criterion (AIC; Sakamoto et al., 1986) and Akaike weight (Wagenmakers and Farrell, 2004). For each region, we calculated the following eight potential models of absolute volume development:

Null, linear, and quadratic age trajectories for the full sample:


$$Y_{ij}=B_{0i}+\epsilon_{ij}$$

$$Y_{ij}=B_{1}\cdot x_{ij}+B_{0i}+\epsilon_{ij}$$

$$Y_{ij}=B_{2}\cdot {x_{ij}}^{2}+B_{1}\cdot x_{ij}+B_{0i}+\epsilon_{ij}$$

Null, linear, and quadratic age trajectories with a main effect of PAE (group):


$$Y_{ij}=\left(B_{3}\cdot P_{ij}+B_{0i}\right)+\epsilon_{ij}$$

$$Y_{ij}=B_{1}\cdot x_{ij}+\left(B_{3}\cdot P_{ij}+B_{0i}\right)+\epsilon_{ij}$$

$$Y_{ij}=B_{2}\cdot {x_{ij}}^{2}+B_{1}\cdot x_{ij}+\left(B_{3}\cdot P_{ij}+B_{0i}\right)+\epsilon_{ij}$$

Linear and quadratic age trajectories with included interactions of PAE and age or age^2^:


$$Y_{ij}=\left(B_{4}\cdot P_{ij}+B_{1}\right)\cdot x_{ij}+\left(B_{3}\cdot P_{ij}+B_{0i}\right)+\epsilon_{ij}$$

$$Y_{ij}=\left(B_{5}\cdot P_{ij}+B_{2}\right)\cdot {x_{ij}}^{2}+\left(B_{4}\cdot P_{ij}+B_{1}\right)\cdot x_{ij}+\left(B_{3}\cdot P_{ij}+B_{0i}\right)+\epsilon_{ij}$$

Where for each region of interest, $Y_{ij}$ = the volume measurement for the *j*th timepoint for the *i*th subject, $x_{ij}$ = subject’s age at time of scan, $P_{ij}$ = group (PAE = 1, unexposed = 0), $B_{0i}$ = subject-specific y-intercept, $B_{1}$ = coefficient for age, $B_{2}$ = coefficient for age^2^, $B_{3}$ = coefficient for group main effect, $B_{4}$ = coefficient for age-by-group interaction, $B_{5}$ = coefficient for age^2^-by-group interaction and $\epsilon_{ij}$ = random error.

Models were calculated using R package lme4 (Bates et al., 2015; R Core Team, 2023). The best fitting model was defined as the one with the lowest AIC value, a measure of out-of distribution prediction error (Sakamoto et al., 1986). For each model, we additionally calculated Akaike weights, a probability between 0 and 1 that a given model is the best at minimizing Kullback-Leibler discrepancy among a set of models (Wagenmakers and Farrell, 2004).

To address potential sex differences, we conducted a second analysis in each group (PAE or unexposed) separately, using the following eight mixed effects trajectory models:

Null, linear, and quadratic age trajectories for the group:


$$Y_{ij}=B_{0i}+\epsilon_{ij}$$

$$Y_{ij}=B_{1}\cdot x_{ij}+B_{0i}+\epsilon_{ij}$$

$$Y_{ij}=B_{2}\cdot {x_{ij}}^{2}+B_{1}\cdot x_{ij}+B_{0i}+\epsilon_{ij}$$

Null, linear, and quadratic age trajectories with an included main effect of sex:


$$Y_{ij}=\left(B_{3}\cdot S_{ij}+B_{0i}\right)+\epsilon_{ij}$$

$$Y_{ij}=B_{1}\cdot x_{ij}+\left(B_{3}\cdot S_{ij}+B_{0i}\right)+\epsilon_{ij}$$

$$Y_{ij}=B_{2}\cdot {x_{ij}}^{2}+B_{1}\cdot x_{ij}+\left(B_{3}\cdot S_{ij}+B_{0i}\right)+\epsilon_{ij}$$

Linear and quadratic age trajectories with included interactions of sex and age or age^2^:


$$Y_{ij}=\left(B_{4}\cdot S_{ij}+B_{1}\right)\cdot x_{ij}+\left(B_{3}\cdot S_{ij}+B_{0i}\right)+\epsilon_{ij}$$

$$Y_{ij}=\left(B_{5}\cdot S_{ij}+B_{2}\right)\cdot {x_{ij}}^{2}+\left(B_{4}\cdot S_{ij}+B_{1}\right)\cdot x_{ij}+\left(B_{3}\cdot S_{ij}+B_{0i}\right)+\epsilon_{ij}$$

Where for each region of interest, $Y_{ij}$ = the volume measurement for the *j*th timepoint for the *i*th subject, $x_{ij}$ = subject’s age at the time of scan, $S_{ij}$ = subject sex (male = 0, female = 1), $B_{1}$ = coefficient for age, $B_{2}$ = coefficient for age^2^, $B_{0i}$ = subject-specific y-intercept, $B_{3}$ = coefficient for sex main effect, $B_{4}$ = coefficient for age by sex interaction, $B_{5}$ = coefficient for age^2^ by sex interaction and $\epsilon_{ij}$ = random error.

It is well-established that mean group differences exist in total brain size between males and females (Paus, 2010). From this biological reality has risen the need to account for total brain size in analyses of sex differences in research on brain volumes (DeCasien et al., 2022). As our primary aim in the study was to characterize trajectories of absolute volume development, our primary analyses did not control for total intracranial volume (ICV). However, given known mean differences in brain volume between males and females and a desire to make our study as comparable as possible to previous and future studies examining both absolute and corrected volumes, we performed a supplementary analysis for within-group sex differences with ICV included as a covariate in the hierarchy of models.

Using the beta values from fitted trajectory models, we computed percent change over time, age at peak volume, and average volume difference between groups (PAE vs. unexposed) and between sexes. We additionally determined partial eta squared (*η*_p_^2^) for all model effects to examine the magnitude of developmental changes in gray matter volume. Magnitude of effect size conveyed by *η*_p_^2^ are typically benchmarked as follows: small = 0.0099, medium = 0.0588, large = 0.1379 (Cohen, 2013).

Our rationale for using Akaike weights, effect sizes, and the calculated development metrics as the primary statistics of interest in our study, as opposed to the more common approach of using *p*-values, is that the primary aim of this study is to thoroughly describe the development trajectories arising from the data, rather than to test a specific hypothesis. The Akaike weights, and specifically Akaike weights >0.7, guide which regions we use as exemplars of patterns we observed across regions as the models in these regions have the strongest support of evidence provided in the data and do not indicate that some regions have “true” effects while other regions do not. The primary results of interest in this study are the development metrics such as percent change or percent difference between groups, which are meant to describe the data rather than prove or disprove a hypothesis.

## 3 Results

### 3.1 Total ICV and total gray matter volume

**Figure 1:**
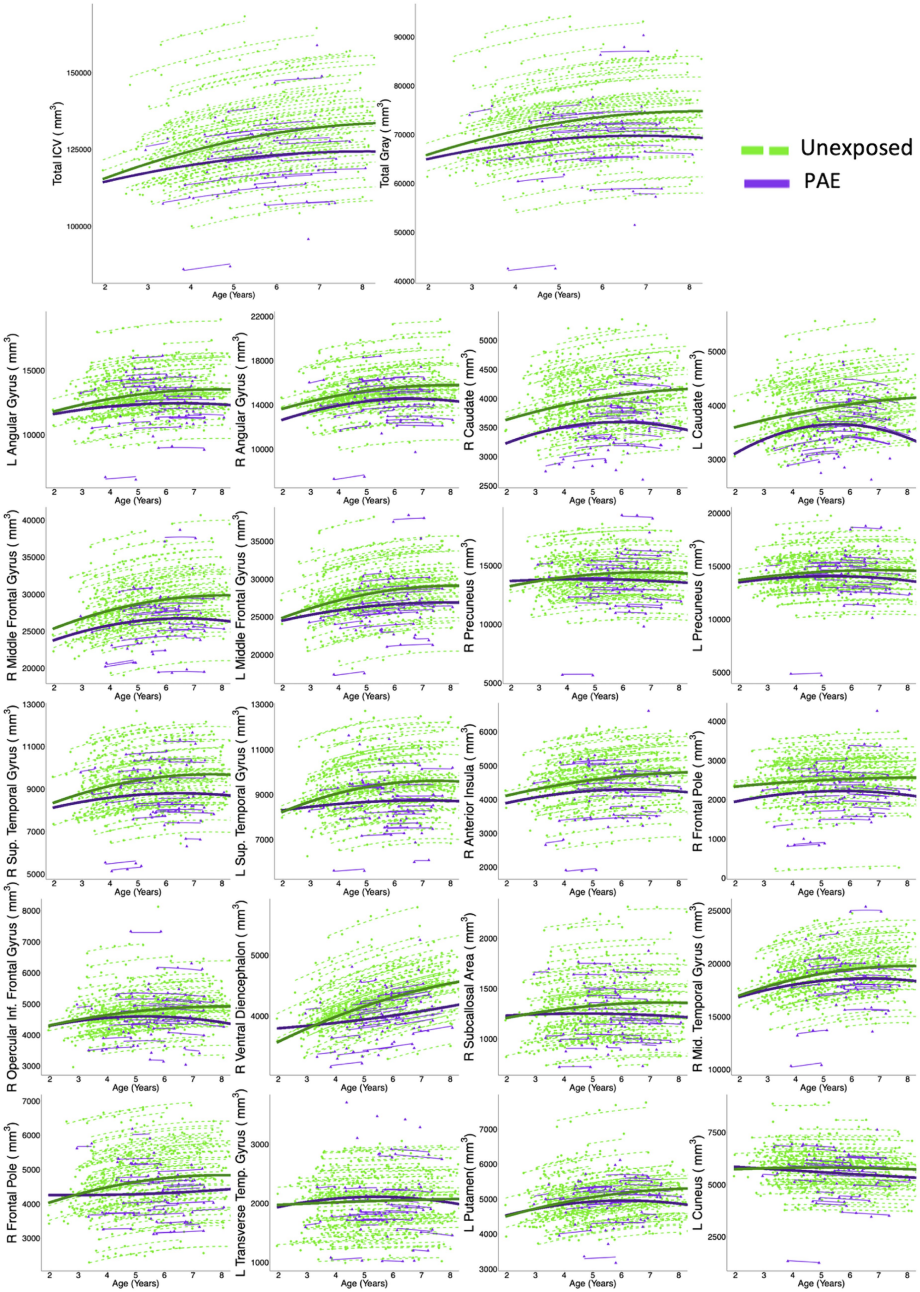
Twenty gray matter regions, plus total ICV and total gray matter, had model trajectories that included group-age interaction terms, and Akaike weights >0.7, indicating strong evidence for model fit. Dots represent a single measurement for a single participant. Thin lines connect volume measurements within the same participant, and thick lines represent group-level trajectories (green = controls; purple = PAE). In general, the PAE group showed fewer age-related changes than controls.

Total ICV and total gray matter volume both followed inverted-u shaped (quadratic) trajectories and were best fit by models including PAE-by-age and PAE-by-age^2^ interactions, with high probabilities of good model fit (ICV: *w* = 0.99, *η*_p_^2^_age*PAE_ < 0.001, *η*_p_^2^_age2*PAE_ < 0.001; total gray: *w* = 0.99, *η*_p_^2^_age*PAE_ = 0.002, *η*_p_^2^_age2*PAE_ < 0.001). Total ICV and total gray matter volume increases were smaller in the PAE group than in the unexposed group, and volumes peaked earlier in the PAE group (ICV: 8.1 years for PAE vs. 9.0 years for unexposed; total gray matter: 6.8 years for PAE vs. 8.2 years for unexposed; Table 2; Figure 1).

**Table 2:** Development metrics for the sample of children with PAE and unexposed children in regions with trajectory models with strong support of evidence (Akaike weight > 0.7).

	Percent change, PAE			Percent change, unexposed			Age at largest volume (years)			
Region	3–4.5 years	4.5–6 years	6–7.5 years	3–4.5 years	4.5–6 years	6–7.5 years	PAE	Unexposed	Difference (unexposed-PAE)	Average percent larger in Unexposed or (PAE)
^††^Total ICV	2.94	1.88	0.88	4.82	3.30	1.94	8.11	9.06	0.95	6.44
^††^Total Gray	2.81	1.40	0.07	4.47	2.84	1.37	6.83	8.22	1.39	6.38
^††^Left angular gyrus	2.84	1.23	−0.31	4.97	2.99	1.22	6.45	7.83	1.39	8.17
^††^Left Caudate	6.87	1.13	−4.12	4.44	3.29	2.25	5.57	8.5^*^	NA	11.94
^††^Left cuneus	−2.23	−2.20	−2.17	0.59	−0.09	−0.78	8.5^*^	5.04	NA	4.76
^†^Left frontal operculum	6.94	4.42	2.25	6.50	3.66	1.17	8.46	7.49	−0.96	8.36
^†^Left lateral orbital gyrus	2.66	2.59	2.53	5.96	3.50	1.33	8.5^*^	7.73	NA	12.68
^††^Left middle frontal gyrus	3.41	2.07	0.84	5.62	3.54	1.70	7.80	8.23	0.43	8.23
^††^Left precuneus	1.77	−0.02	−1.78	2.62	1.37	0.18	5.23	6.99	1.75	4.22
^††^Left Putamen	3.76	1.31	−0.99	5.46	3.67	2.09	6.10	8.5^*^	NA	4.81
^††^Left superior temporal gyrus	2.03	1.06	0.13	5.92	3.32	1.02	6.96	7.44	0.48	10.29
^††^Left transverse temporal gyrus	−1.01	−1.02	−1.03	1.14	1.12	1.11	2.5^*^	8.5^*^	NA	(2.59)
^†^Left triangular part of the inferior frontal gyrus	5.35	3.00	0.90	5.05	3.15	1.44	7.42	8.10	0.68	9.61
^††^Right angular gyrus	5.69	2.57	−0.24	5.34	3.24	1.37	6.62	7.92	1.30	8.79
^††^Right anterior insula	1.11	1.49	1.85	6.79	4.25	2.06	8.5^*^	8.28	NA	10.20
^††^Right Caudate	1.46	0.47	−0.50	1.29	0.46	−0.35	8.5^*^	8.5^*^	NA	14.04
^††^Right frontal pole	2.19	2.40	2.60	5.24	3.48	1.91	8.5^*^	8.5^*^	NA	11.04
^†^Right gyrus rectus	3.12	2.16	1.26	5.74	3.37	1.27	2.70	8.00	5.30	10.08
^†^Right medial orbital gyrus	4.22	2.55	1.02	5.37	3.51	1.86	8.5^*^	8.00	NA	8.99
^††^Right middle frontal gyrus	4.78	2.13	−0.30	5.84	3.64	1.69	7.64	7.34	−0.30	11.57
^††^Right middle temporal gyrus	3.99	1.80	−0.23	5.50	3.37	1.47	5.65	6.86	1.21	5.69
^††^Right opercular part of the inferior frontal gyrus	2.86	0.14	−2.49	4.75	2.99	1.42	8.5^*^	8.5^*^	NA	6.33
^††^Right posterior insula	5.60	1.21	−2.84	3.23	2.15	1.14	6.01	7.03	1.02	14.64
^††^Right precuneus	0.43	−0.32	−1.07	3.36	1.68	0.11	NA	8.5^*^	NA	4.25
^††^Right subcallosal area	0.63	−0.39	−1.41	4.48	2.58	0.86	6.67	8.5^*^	NA	8.55
^††^Right superior temporal gyrus	3.23	1.42	−0.28	5.57	3.22	1.13	2.5^*^	8.5^*^	NA	10.33
^†^Right triangular part of the inferior frontal gyrus	4.30	2.90	1.62	4.80	2.93	1.25	2.5^*^	8.5^*^	NA	10.81
^††^Right Ventral Diencephalon	1.97	2.39	2.77	7.43	5.67	4.19	8.5^*^	7.92	NA	8.35

Percent change was calculated from the trajectory models with PAE-by-age and/or PAE-by-age^2^ interactions. Average percent larger was calculated from the models with only a main effect of PAE. Some values for age at largest volume have been manually imputed as either 2.5 years, indicating that the volume trajectory only decreased between the observed 2 and 8 years, or 8.5 years, indicating that volume only increased; imputed ages are marked with^*^. ^††^Denotes that the region’s best-fitting trajectory model had a PAE-by-age and/or PAE-by-age^2^ interactions and Akaike weight >0.7, while ^†^indicates that the best fitting trajectory model included only a main effect of PAE and had an Akaike weight >0.7.

### 3.2 Regional gray matter development

**Figure 2:**
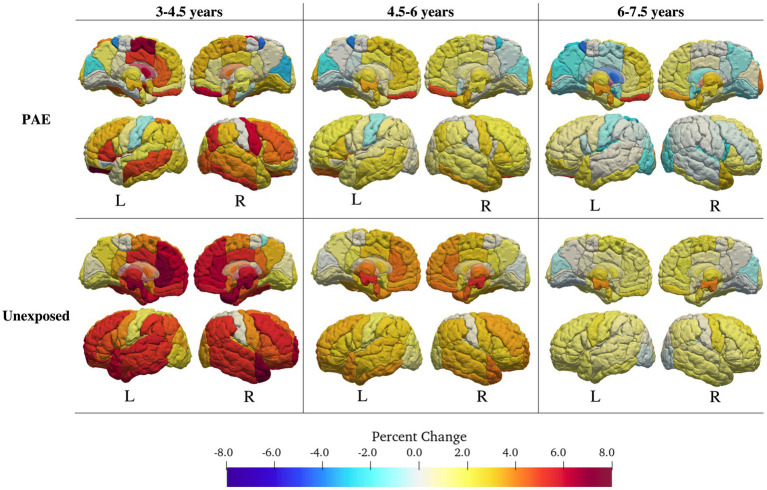
Volume changes for the PAE group (top) and the control group (bottom). Change in volume was calculated for intervals of 1.5 years (3–4.5; 4.5–6, 6–7.5 years) using the best fitting (lowest AIC value) interaction model for each region. The PAE group showed fewer age-related changes in volume than the control group, and earlier decreases in volume.

Fifty-nine of 116 regions were best fit by a model including both a main effect of PAE and a PAE-by-age^2^ and/or PAE-by-age interaction term (Table 2; Figure 2; Supplementary Tables S1, S2). Of these, 20 regions had high Akaike weights, 24 had medium weights, and 15 had low weights. In regions with high Akaike weights, effect sizes for age-by-PAE and age^2^-by-PAE interactions were small Regardless of the strength of the Akaike weight, the PAE group showed less overall volume growth and earlier peaks than the unexposed group. In general, the unexposed group showed rapid gray matter growth in frontal and temporal regions from ages 3 to 4.5 years and moderate growth from ages 4.5 to 7.5, while the PAE group showed more limited early gray matter growth, and more pronounced volume decreases between ages 6 and 8 years.

**Figure 3:**
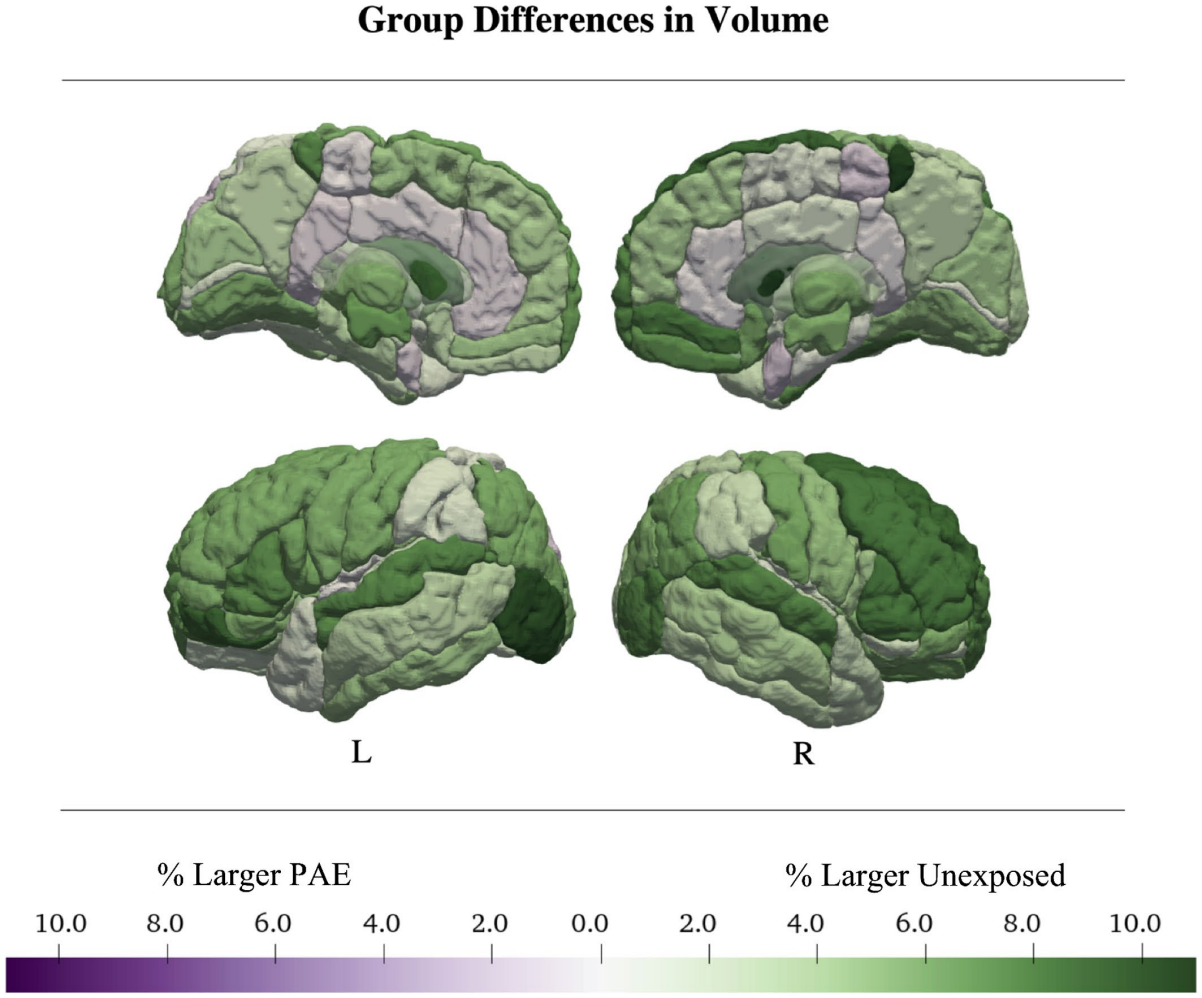
Total and regional gray matter volumes were larger in the control group. Regional volume was up to 11% larger in controls than in the PAE sample. Average group differences in volume were calculated from the main-effects only models.

Thirty-two regions were best fit by a model including a main effect of PAE with no interactions; 6 regions had high akaike weights (> 0.7), 16 medium (> 0.5), and 10 low (< 0.5). Brain regions with models with Akaike weights above 0.7 included the bilateral triangular inferior frontal gyrus (right: *w* = 0.85, *η*_p_^2^*_PAE_* = 0.05; left: *w* = 0.81, *η*_p_^2^*_PAE_* = 0.043), right gyrus rectus (*w* = 0.72, *η*_p_^2^*_PAE_* = 0.029), medial orbital gyrus (*w* = 0.71, η_p_^2^_PAE_ = 0.036), the left lateral orbital gyrus (*w* = 0.72, *η*_p_^2^*_PAE_* = 0.04), and frontal operculum (*w* = 0.7, *η*_p_^2^*_PAE_* = 0.027). In all of these regions, the unexposed group had a larger volume than the PAE group (Table 2; Figure 3; Supplementary Tables S1, S2).

The remaining 25 regions were best fit by models without any PAE term, only describing either linear (2 regions) or quadratic (23 regions) volume changes over time, with no group differences.

### 3.3 Sex differences

In children with PAE, total ICV was best fit by a linear model with a main effect of sex; (*w* = 0.28, *η*_p_^2^*_Sex_* = 0.24) males had 11.8% larger volume than females. Total gray matter volume was best fit by a quadratic model with sex-by-age and sex-by-age^2^ interactions (*w* = 0.4, *η*_p_^2^_age*Sex_ = 0.01, *η*_p_^2^_age2*Sex_ = 0.004) where males had larger volume increases than females, as well as 12% larger total gray matter volumes than females (Table 3; Supplementary Tables S3, S4).

**Table 3:** Development metrics for the mal and female children with PAE in regions with trajectory models with strong support of evidence (Akaike weight > 0.7).

	Percent change, PAE male			Percent change, PAE female			
Region	3–4.5 years	4.5–6 years	6–7.5 years	3–4.5 years	4.5–6 years	6–7.5 years	Average percent larger in male or (female)
^†^Right lingual gyrus	3.46	1.09	−1.16	3.06	1.10	−0.75	16.59
^††^ Right superior temporal gyrus	5.78	1.67	−2.08	1.42	1.60	1.77	9.81

Percent change was calculated from the trajectory models with sex-by-age and/or sex-by-age^2^ interactions. Average percent larger was calculated from the models with only a main effect of sex. ^††^Denotes that the region’s best-fitting trajectory model had a sex-by-age and/or sex-by-age^2^ interactions and Akaike weight >0.7, while ^†^indicates that the best fitting trajectory model included only a main effect of sex and had an Akaike weight >0.7.

In the unexposed group, total ICV was best fit by a quadratic model without a sex term (*w* = 0.39). Total gray matter volume was best fit by a quadratic model with a main effect of sex (*w* = 0.58, *η*_p_^2^*_Sex_* = 0.01) but no interaction; males had 2.5% larger total gray matter volume than females (Table 4; Supplementary Tables S5, S6).

**Table 4:** Development metrics for male and female children in the unexposed sample in regions with trajectory models with strong support of evidence (Akaike weight > 0.7).

	Percent change, unexposed male			Percent change, unexposed female			
Region	3–4.5 years	4.5–6 years	6–7.5 years	3–4.5 years	4.5–6 years	6–7.5 years	Average percent larger in male or (female)
^††^Left amygdala	8.29	4.67	1.61	7.02	4.06	1.49	5.45
^††^Left anterior cingulate gyrus	8.07	5.09	2.58	5.06	3.78	2.65	7.68
^†^Left anterior insula	5.07	3.14	1.40	4.86	2.84	1.01	1.64
^††^Left cuneus	1.19	0.08	−1.02	−0.05	−0.28	−0.52	3.99
^†^Left entorhinal area	6.95	4.69	2.75	7.06	4.69	2.66	9.43
^††^Left middle cingulate gyrus	5.69	3.60	1.75	5.68	3.52	1.60	6.07
^†^Left middle frontal gyrus	3.85	2.09	0.46	3.28	1.65	0.12	3.07
^††^Left posterior cingulate gyrus	3.75	2.38	1.12	2.18	2.01	1.85	7.48
^†^Left subcallosal area	6.01	3.85	1.95	4.82	3.47	2.26	3.10
^†^Left temporal pole	6.68	4.46	2.54	4.74	3.83	3.01	1.25
^††^Left thalamus proper	5.95	3.72	1.76	7.16	4.18	1.62	7.44
^†^Right anterior insula	8.11	4.55	1.52	7.02	4.54	2.41	8.28
^†^Right basal forebrain	5.55	3.13	0.97	5.92	3.67	1.69	4.60
^†^Right frontal operculum	6.91	4.65	2.72	7.16	4.59	2.40	5.66
^†^Right anterior cingulate gyrus	5.85	3.74	1.89	5.86	3.77	1.94	0.69
^†^Right medial frontal cortex	5.84	3.09	0.65	5.83	3.29	1.04	8.39
^†^Right middle cingulate gyrus	6.07	3.65	1.51	5.73	3.67	1.85	6.83
^†^Right middle frontal gyrus	3.97	2.08	0.33	4.10	1.91	−0.12	4.40
^††^Right parietal operculum	4.90	3.10	1.48	4.61	3.13	1.79	3.86
^††^Right Putamen	2.72	1.17	−0.31	2.08	0.84	−0.37	2.91
^†^Right superior frontal gyrus	4.64	2.75	1.04	4.46	2.45	0.61	7.19

Percent change was calculated from the trajectory models with sex-by-age and/or sex-by-age^2^ interactions. Average percent larger was calculated from the models with only a main effect of sex. ^††^Denotes that the region’s best-fitting trajectory model had a sex-by-age and/or sex-by-age^2^ interactions and Akaike weight >0.7, while ^†^indicates that the best fitting trajectory model included only a main effect of sex and had an Akaike weight >0.7.

Among the four subsamples (unexposed males, unexposed females, males with PAE, females with PAE), unexposed males had the largest peak gray matter volume (755,907 mL), then unexposed females (97.8% of unexposed male volume; 739,015 mL), followed by males with PAE (97.4% of unexposed male volume; 735,972 mL), and lastly females with PAE (86.7% of unexposed male volume; 655,570 mL; Tables 3, 4; Supplementary Tables S3–S6).

**Figure 4:**
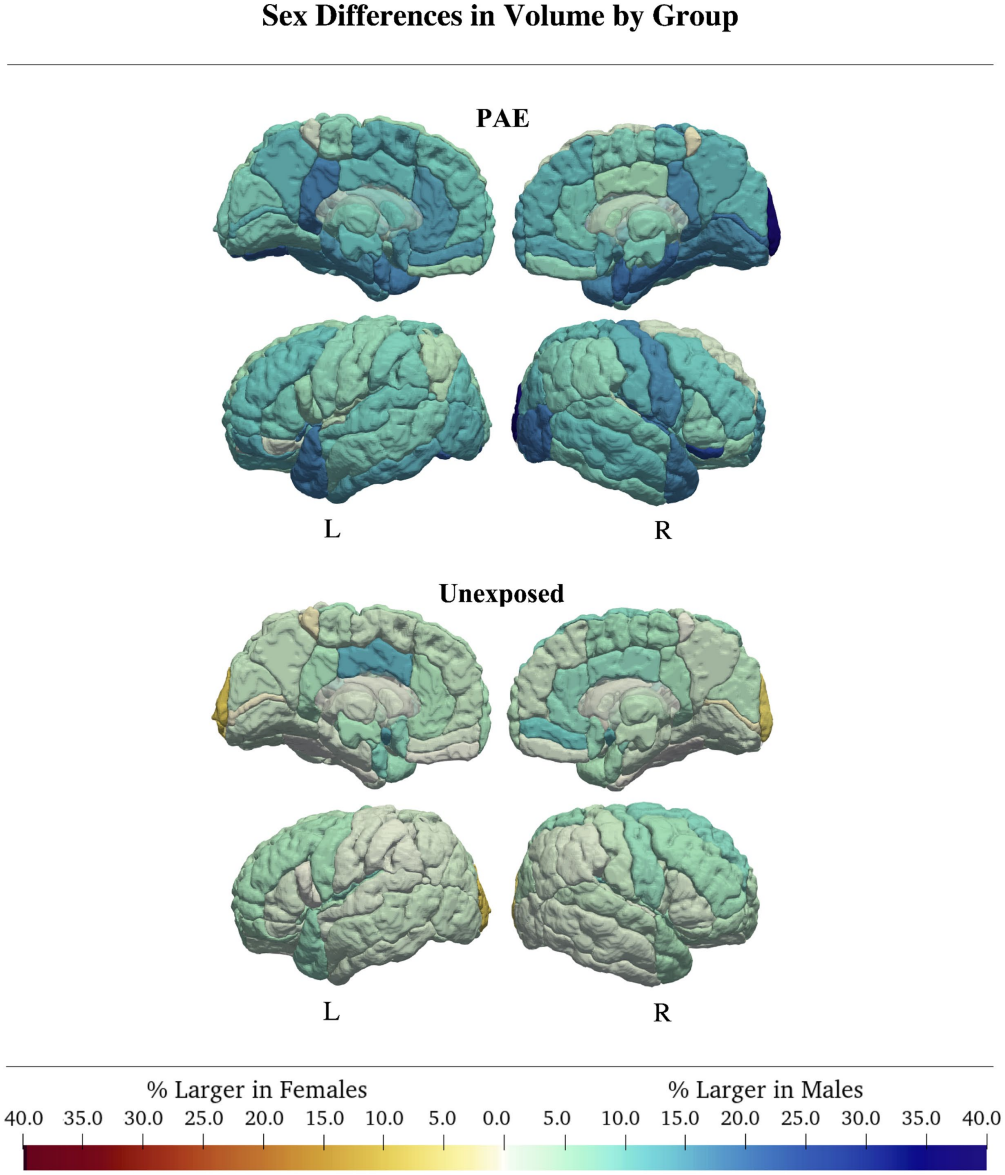
In both groups, males generally had larger volumes than females. Group differences were more pronounced in the PAE group, where males had regional volumes up to 40% larger than females, compared to controls, where sex differences were generally <5%.

In children with PAE, 24 of 116 regions were best fit by a model with a sex-by-age or sex-by-age^2^ interaction. Of these, only one region (right superior temporal gyrus) had an Akaike weight greater than 0.7 (*w* = 0.83). Here, males showed a strong curvilinear pattern and females a more linear trajectory (*η*_p_^2^_age*sex_ = 0.12, *η*_p_^2^_age2*sex_ = 0.15). Eight other regions had an Akaike weight between 0.5–0.7, and 15 had Akaike weights <0.5. These other regions with interactions followed a similar pattern to the right superior temporal gyrus, with more age-related changes in males than females. The region with the largest sex discrepancy was the left amygdala (*w* = 0.54), where males showed an average 4% increase in volume/year in contrast to volume decreases of 1.7%/year in females (*η*_p_^2^_age*sex_ = 0.07; Figure 4; Table 3; Supplementary Tables S3, S4).

For the unexposed group, 27 of 116 brain regions were best fit by a model with a sex-by-age or sex-by-age^2^ interaction. Akaike weights were > 0.7 for the right parietal operculum and putamen, and the left anterior cingulate gyrus, amygdala, cuneus, middle cingulate gyrus, posterior cingulate gyrus, posterior insula, and thalamus, where males had slightly more age-related changes than females. Interaction effect sizes were small (*η*_p_^2^_age*sex_ = 0.02, *η*_p_^2^_age2*sex_ = 0.01). Similar to the PAE group, the left amygdala had the largest discrepancy in development rate between males and females in the unexposed group with males showing an average 3.3% increase/year while females showed an average 2.7% increase/year (*η*_p_^2^_age*sex_ = 0.02, *η*_p_^2^_age2*sex_ = 0.01). Of the remaining regions, 13 had Akaike weights between 0.5–0.7 and five had Akaike weights <0.5. Similar patterns of less overall change in females were observed in these areas (Table 3; Supplementary Tables S4, S5).

In children with PAE, there was a main effect of sex in most (66/116) regions. However, only the right lingual gyrus had an Akaike weight > 0.7 (*w* = 0.75, *η*_p_^2^*_Sex_* = 0.16), where males had 16.6% larger volumes than females. Four of 116 regions had Akaike weights between 0.5–0.7, and 61/116 regions had an Akaike weight < 0.5. Across all regions with a main effect of sex, males had larger volumes by an average of 13.7% (Figure 4; Table 3; Supplementary Tables S3, S4).

In the unexposed group, 54 of 116 gray matter regional trajectories were best fit by a model with only a main effect of sex, no interactions. Of these regions, 13/54 regional models had an Akaike weight > 0.7, 19/54 regions had an Akaike weight < 0.5, and 21/54 regions had Akaike weights between 0.5–0.7. In regions with high support (Akaike weight > 0.7,) males had 7–12% larger volumes than females and effect sizes were generally small (Figure 4; Table 4). This pattern was representative of most remaining regions with sex main effects, except the right and left occipital pole, which were 7 and 8.9% larger in females, respectively (*w*_right_ = 0.37; *w*_left_ = 0.47; Figure 4; Table 4; Supplementary Tables S5, S6).

Our supplementary analysis of sex differences with ICV as a covariate told a similar story to the primary analysis, although the number of regions best fit by models with interaction and/or main effects of sex were reduced. Regions with the greatest evidence and largest group differences for age^2^-by-sex and age-by-sex interactions and/or sex main effects such as the left amygdala showed similar patterns whether controlling for ICV in the model or not. Notably, Akaike weights were generally smaller for models which controlled for ICV, indicating that the weight of evidence in support of models with ICV as a covariate was lower than evidence supporting models without ICV as a covariate. Specifically, for the exposed group, no best fitting model exceeded an Akaike weight of 0.6, and in the unexposed group, only 9 regional models had Akaike weights >0.7 as compared to 22 regional models when ICV was not included.

## 4 Discussion

Here, we show in a longitudinal study, that young children with PAE generally have less overall age-related gray matter volume changes and earlier volume peaks than unexposed children. These findings are consistent with those in older children (Lebel et al., 2011; Donald et al., 2015), suggesting that PAE leads to reduced brain plasticity and earlier brain maturation. Additionally, we observed more pronounced sex differences in volume in the PAE group than in the unexposed group, with females with PAE having especially reduced gray matter volumes.

Our cohort of young children with PAE showed attenuated age-related gray matter volume changes as compared to unexposed children. This pattern is consistent with the few previous longitudinal research on gray matter volume in older children (Lebel et al., 2012; Treit et al., 2013; Hendrickson et al., 2018). However, prior studies found altered gray matter trajectories in more specific cortical regions such as the occipital and parietal lobes (Lebel et al., 2012), or the right hemisphere (Hendrickson et al., 2018). In contrast, our results seem to suggest widespread alterations in gray matter trajectories. This may be due, in part, to different statistical approaches, with this study relying on AIC values and weights (Wagenmakers and Farrell, 2004) to determine relevant trends, rather than *p*-values. Due to the different statistical approaches, it is likely that previous research may have under-estimated the extent of regional differences in gray matter volume. As development metrics such as rate of change, percent difference between groups, or identifying differences with the largest effect sizes may have more clinical relevance than statistically significant effects alone, we suggest that future research seeking to map brain development trajectories aim to holistically report developmental metrics across all regions examined, rather than focusing solely on statistically significant findings. Our findings are also consistent with measures of white matter microstructure in an overlapping sample of children, where mean diffusivity (a measure of water movement from diffusion tensor imaging) showed smaller overall decreases in the PAE group than in the unexposed group (Kar et al., 2022). Taken together, these findings suggest that alterations in development trajectories associated with PAE are widespread during early childhood.

Young children with PAE had smaller total and regional gray matter volumes than unexposed children. This is consistent with the larger body of literature on brain structure in individuals with PAE, indicating global reductions in gray matter, white matter, and total gray matter volume across infants, older children, adolescents, and adults (Lebel et al., 2011; Donald et al., 2015, 2016). The age range of our study connects previous findings in neonates (Donald et al., 2016) to the well-established findings in older children and adolescents (Mattson et al., 1996; Archibald et al., 2001; Cortese et al., 2006; Willoughby et al., 2008; Astley et al., 2009; Nardelli et al., 2011; Roussotte et al., 2011; Chen et al., 2012; Treit et al., 2013; Rajaprakash et al., 2014). Regional gray matter volumes were up to 11% smaller in PAE as compared to the unexposed group. While the majority of previous studies have not reported the specific percent reduction gray matter volume in PAE, our observations are comparable to Treit et al. (2017), who reported group differences between 7 and 17% depending on region.

Sex differences were more pronounced in children with PAE than in the unexposed group, which was true for both main effects (i.e., overall group differences in volume) as well as sex-by-age and/or sex-by-age^2^ interactions. In the unexposed group, rates of development were similar for males and females, and even in regions with a sex-age interaction, differences between sexes were very small (*η*_p_^2^ < 0.06). In contrast, development rates differed more starkly between males and females with PAE, where females showed consistently smaller age-related changes. We found pronounced sex differences in development rates of the left amygdala, with females in the PAE group showing the smallest rate of volume increase as compared to any other group. While we are not aware of any studies which have specifically addressed sex differences in amygdala volume in children with PAE, the amygdala is known to be affected by PAE (Lebel et al., 2011) and children of either sex may be differentially vulnerable to the effects of PAE (McCarthy, 2016). Recently amygdala volume was also been shown to be related to internalizing symptoms in children (Sammallahti et al., 2023). As females with PAE are more likely to experience internalizing problems than males (Flannigan et al., 2023), this early discrepancy in amygdala development rates may hint at the neural underpinnings of later outcomes. Another area with large differences between male and female children with PAE was the right lingual gyrus, which was 16.6% larger in males. Previous research has indicated that the right lingual gyrus shows altered development of cortical structure, such as cortical thinning (Gimbel et al., 2023) in individuals with PAE as compared to an unexposed group. While we are not aware of any other studies that have specifically examined sex effects on development of the right lingual gyrus, it may be that male and female children are differentially susceptible to the insult of PAE, manifesting in stark differences in gray matter regions that are prominently affected by PAE.

While prior studies have also reported larger sex differences associated with PAE, they tended to find males with PAE to be more affected than females (Treit et al., 2017; Subramoney et al., 2022). Males have often been regarded as more vulnerable to adverse exposures, which may stem from chromosomal, endocrine, and inflammatory differences (McCarthy, 2016). This has been supported by observations in MRI studies that males with PAE show greater differences in brain structure compared to an unexposed group than their female counterparts (Treit et al., 2017; Subramoney et al., 2022). However, not all previous studies have indicated more pronounced sex differences in alcohol exposed versus unexposed samples; Our findings contrast with the study by Nardelli et al. (2011), which found that brain volumes in male and female children with PAE were similarly reduced, and that larger sex differences existed in the unexposed group. The sample in that study was both cross-sectionally sampled and fairly small (28 youth with PAE), which may have made detecting certain sex effects difficult; future replications of findings will help to elucidate a more consistent picture of sex differences in children with PAE.

The direction of sex differences in children with PAE may depend on the age of the sample examined. In a study with a wider age range of primarily older children through young adults, volume differences between youth with PAE and unexposed controls were greater in males (Treit et al., 2017). In a very young sample of 2–3 year-olds, a similar pattern was shown: males with PAE had significant volume reductions in the putamen as compared to unexposed males, while females with and without PAE had similar volumes (Subramoney et al., 2022). Additionally, while it considered gender rather than sex, Lebel et al. (2012) showed that unexposed boys/men had more curved trajectories in precentral, supramarginal, and superior parietal cortical regions than the other three groups. Sample size, age, and quality control procedures can also affect the shape of development trajectories, leading to different conclusions from similar-but-different data (Fjell et al., 2010; Ducharme et al., 2016). In summary, the nature of observed effects may vary based on the age range of the observed sample, strictness of quality control procedures, as well as whether sex or gender was used in the analysis.

Sex differences specifically must be interpreted with caution, as differential brain structure between males and females does not necessarily reflect differences in cognition and behavior (DeCasien et al., 2022). Indeed, sex differences in structural brain trajectories may instead indicate that males and females have different developmental windows of opportunity (Andersen, 2003), with implications for interventions supporting healthy brain development and skill acquisition. Our finding of greater differences from the unexposed group in females with PAE may reflect developmental differences present in early childhood, and sex differences may present differently at various life stages.

The altered development trajectories observed in this study may indicate reduced neuroplasticity in young children with PAE. PAE has direct effects on cellular and molecular mechanisms related to plasticity, including receptor function and maturation of microglia (Fontaine et al., 2016; Wilhelm and Guizzetti, 2016), with possible consequences for plasticity (Tooley et al., 2021). Neuroplasticity broadly decreases with age and maturity, which has been well characterized in animal models (Bonfanti and Charvet, 2021; Tooley et al., 2021). The earlier onset of volume decreases in children with PAE is consistent with a profile of earlier maturation, and perhaps an accompanying accelerated decrease in developmental plasticity. Accelerated maturation may also mean less time spent in developmental windows of increased plasticity, where the brain can learn and adapt more easily (Tooley et al., 2021). Indeed, research in animal models has indicated that prenatal exposure to ethanol impacts brain structure related to plasticity such as altered dendritic structure of medium spiny neurons (Rice et al., 2012), and pyramidal neurons (Cui et al., 2010; Louth et al., 2018) with downstream effects on behavior (Hamilton et al., 2010). Furthermore, translational studies of MRI in animal models of PAE and FASD have confirmed the macrostructural changes observed in humans that may ultimately indicate altered brain plasticity (Wang and Kroenke, 2015). However, further translational research is necessary to connect findings from *in-vivo* human studies with findings from model systems (Charvet, 2020) including the development of relevant clinical markers of alcohol exposure and how macrostructural changes observed with MRI relate to microstructural alterations such as dendritic spine arborization and synaptic structure. Moreover, many other factors, such as the timing and amount of PAE, the postnatal environment, and genetics, may have compounding effects on brain development trajectories and plasticity in children with PAE (Lebel et al., 2019).

Postnatal environmental factors such as low socioeconomic status, home instability, and adverse experiences can also affect patterns of structural brain development and lead to accelerated maturation (Callaghan and Tottenham, 2016; Tooley et al., 2021). Not only are these types of adverse postnatal exposures more common for children with PAE (Lebel et al., 2019; Holman and Raineki, 2023), but there are also differences in the types of adversity experienced by male and female children with PAE (Flannigan et al., 2021, 2023), adding to the complexity. Evidence for patterns of postnatal influences on brain development in children with PAE remains somewhat scarce. Andre et al. (2020) showed different profiles of cortical volume for children with PAE who had no adverse postnatal exposure than for children with PAE and adverse postnatal exposure (Andre et al., 2020). However, other studies suggest minimal additive effects of postnatal environment For example, one study suggested that the effects of PAE on the brain may not be further influenced by socioeconomic status (Uban et al., 2020) and in a separate multi-risk cohort, PAE was determined to be the dominant risk factor underlying adverse outcomes (Hemingway et al., 2020). Consideration of adverse postnatal exposures, especially as they relate to sex, is needed in future longitudinal studies of PAE provided adequate sample sizes are available.

It remains unclear whether deviations in brain development are directly related to behavior or cognitive difficulties in children with PAE. Previous research has indicated that deviations from typical gray matter development are associated with symptom severity in autistic children (Ecker et al., 2010; Tunç et al., 2019) and children with ADHD (Shaw et al., 2006). However, deviations resulting from teratogenic alcohol exposures may not neatly equate to functional or behavioral challenges and further research is needed to understand whether deviations in brain structural development are directly related to symptom severity in children with PAE (Hanlon-Dearman et al., 2020).

While this study provides an important characterization of gray matter development in a novel sample, it is also subject to some limitations. Many children in the PAE group had co-occurring prenatal exposures and postnatal adverse experiences, which were not accounted for in our analysis. Indeed, it is known that prenatal exposure to drugs such as cocaine, opioids, and stimulants, affect the structure of the developing brain (Conradt et al., 2019; Dufford et al., 2021; Merhar et al., 2021; Radhakrishnan et al., 2021; Sanjari Moghaddam et al., 2021; Balalian et al., 2023; Castro et al., 2023). However, it has been suggested that among concurrent prenatal substance exposures, alcohol explains the most variance in brain outcomes (Hemingway et al., 2020). While PAE may be a primary determinant of brain alterations, considering all prenatal exposures will offer a fuller and more accurate description of brain outcomes, and may help to address some of the variability in brain outcomes observed in children with PAE. Future longitudinal studies with adequate statistical power should consider the myriad of factors that can potentially moderate brain outcomes in children with PAE. Additionally, we did not have complete information about the timing, frequency, or amount of alcohol exposure during pregnancy, as this is often difficult to obtain retrospectively. Information on facial morphology, which has been associated with the severity of brain and cognitive outcomes (Lebel et al., 2011; Roussotte et al., 2011) and provide implicit information regarding the timing of exposure (Sulik, 2005; Bandoli et al., 2022), was also not obtained as part of this study. Associations between neurodevelopmental and behavioral outcomes and PAE appear to be dose and timing-dependent (Sood et al., 2001; O’Leary et al., 2010; Marquardt and Brigman, 2016; Subramoney et al., 2018), so this will be important to examine in future studies that are able to obtain more detailed information. Another limitation of our PAE sample is the somewhat sparse sampling and short inter-scan interval as compared to the relatively large and highly sampled unexposed group. The approximately 8 month average inter-scan interval in the PAE group combined with the average 2 scans per subject results in a limited ability to detect developmental change across the age span. However, it should not be understated that longitudinal MRI samples of young children with PAE are rare, and our study contributes valuable information about brain development in an under-studied population. The exposed sample in this study is also limited in its relatively small size, particularly when split by sex. Individuals with PAE are a highly heterogeneous group, and it is unlikely that our sample of 42 subjects captures the complete range of brain development patterns present across this population. However, small sample sizes are typically of concern in studies where statistical significance of an effect is the primary tool for evaluating the data (Sullivan and Feinn, 2012; Dunkler et al., 2020). Our study, on the other hand, was not reliant on statistical significance and sought instead to characterize development metrics of potential clinical relevance instead. While future research will benefit from larger sample sizes which can fully capture the diversity of development trajectories within the population of individuals with PAE and more robustly capture the nature of sex effects, our descriptive study provides novel insights into potential effects of sex and development in PAE. Lastly, children with PAE who also had a diagnosis of ADHD, learning disabilities, language delays, and mental health diagnoses were included in this study as these diagnoses commonly co-occur with PAE. While it is possible that PAE is the cause of these diagnoses, symptoms related to these above diagnoses are known to have moderate-to-high heritability (Brikell et al., 2021; Georgitsi et al., 2021; Kendall et al., 2021; Morgan et al., 2024), and we are unable to rule out genetic factors which could have caused or contributed to these diagnoses. When possible, future studies should consider heritability of these diagnoses by obtaining information on medical, educational, and mental health histories of biological parents.

## 5 Conclusion

The present study shows altered global and regional gray matter development trajectories in young children with PAE, where PAE is associated with reduced overall volume growth and earlier age-related volume decreases. We also found more pronounced sex differences in children with PAE, suggesting that gray matter volume development in females is especially atypical. Reduced age-related changes in volume may indicate reduced brain plasticity in children with PAE and point to the importance of early recognition and intervention. This study connects previous research on neonates, older children, and adolescents, with PAE and helps understand potential brain mechanisms underlying differential outcomes in individuals with PAE.

## Data availability statement

The datasets presented in this study can be found in online repositories. The names of the repository/repositories and accession number(s) can be found here: https://osf.io/axz5r/.

## Ethics statement

The studies involving humans were approved by Conjoint Health Research Ethics Board at the University of Calgary. The studies were conducted in accordance with the local legislation and institutional requirements. Written informed consent for participation in this study was provided by the participants’ legal guardians/next of kin.

## Author contributions

ML: Conceptualization, Writing – original draft, Writing – review & editing, Data curation, Formal analysis, Methodology. PK: Data curation, Writing – review & editing. NF: Writing – review & editing, Methodology, Supervision. BL: Methodology, Writing – review & editing, Software. WG: Writing – review & editing, Conceptualization. CT: Conceptualization, Writing – review & editing. CM: Conceptualization, Writing – review & editing, Supervision. YH: Writing – review & editing, Methodology, Software. CL: Writing – review & editing, Conceptualization, Funding acquisition, Project administration, Supervision, Writing – original draft.
